# Real-World Use of Do-It-Yourself Artificial Pancreas Systems in Children and Adolescents With Type 1 Diabetes: Online Survey and Analysis of Self-Reported Clinical Outcomes

**DOI:** 10.2196/14087

**Published:** 2019-07-30

**Authors:** Katarina Braune, Shane O'Donnell, Bryan Cleal, Dana Lewis, Adrian Tappe, Ingrid Willaing, Bastian Hauck, Klemens Raile

**Affiliations:** 1 Department of Paediatric Endocrinology and Diabetes Charité - Universitätsmedizin Berlin Berlin Germany; 2 The Insight Centre for Data Analytics University College Dublin Belfield Ireland; 3 Diabetes Management Research Steno Diabetes Center Copenhagen Gentofte Denmark; 4 OpenAPS Seattle, WA United States; 5 AndroidAPS Vienna Austria; 6 #dedoc° Diabetes Online Community Berlin Germany

**Keywords:** artificial pancreas, do it yourself, open source, mobile health, diabetes, type 1 diabetes, pediatric diabetes, closed loop, automated insulin delivery

## Abstract

**Background:**

Patient-driven initiatives have made uptake of Do-it-Yourself Artificial Pancreas Systems (DIYAPS) increasingly popular among people with diabetes of all ages. Observational studies have shown improvements in glycemic control and quality of life among adults with diabetes. However, there is a lack of research examining outcomes of children and adolescents with DIYAPS in everyday life and their social context.

**Objective:**

This survey assesses the self-reported clinical outcomes of a pediatric population using DIYAPS in the real world.

**Methods:**

An online survey was distributed to caregivers to assess the hemoglobin A_1c_ levels and time in range (TIR) before and after DIYAPS initiation and problems during DIYAPS use.

**Results:**

A total of 209 caregivers of children from 21 countries responded to the survey. Of the children, 47.4% were female, with a median age of 10 years, and 99.4% had type 1 diabetes, with a median duration of 4.3 years (SD 3.9). The median duration of DIYAPS use was 7.5 (SD 10.0) months. Clinical outcomes improved significantly, including the hemoglobin A_1c_ levels (from 6.91% [SD 0.88%] to 6.27% [SD 0.67]; *P*<.001) and TIR (from 64.2% [SD 15.94] to 80.68% [SD 9.26]; *P*<.001).

**Conclusions:**

Improved glycemic outcomes were found across all pediatric age groups, including adolescents and very young children. These findings are in line with clinical trial results from commercially developed closed-loop systems.

## Introduction

Over 30 years ago, the Diabetes Control and Complications Trial showed benefits of intensive diabetes management in delaying the onset and reducing the severity of diabetes-related complications [1]. People diagnosed at a young age are particularly at risk for developing long-term complications and comorbidities during childhood and later throughout life. Owing to this, therapeutic guidelines recommend tight glycemic control, with a target hemoglobin A_1c_ (HbA_1c_) level<7.0% (53 mmol/mol) for all people with diabetes [2]. For children, adolescents, and young adults, guidelines even recommend the lowest achievable HbA_1c_ without undue exposure to severe hypoglycemia, balanced with quality of life and burden of care [3]. Today, despite significant advances in therapy and technological developments, only 17% of all children and adolescents with diabetes achieve an HbA_1c_ level<7.5% (58 mmol/mol) [4].

Multiple clinical trials have shown that closed-loop insulin delivery systems (also known as automated insulin delivery systems or “artificial pancreas”) designed for commercial use are safe and effective in reducing hyper- and hypoglycemia in people of all age groups with diabetes, including adolescents and children [5-9]. Closed-loop systems are characterized by automated insulin delivery in response to the user’s glucose level. Although commercial systems are under development and some have recently become available in a limited number of countries, they are not universally available, accessible, or affordable. Behind the hashtag #WeAreNotWaiting, a community of people with diabetes and their families have created new tools and systems, in addition to the existing, already approved medical devices, and shared them via open source platforms in order to help others with diabetes better utilize their devices and data. One of the most significant innovations to emerge through this movement is the Do-it-Yourself Artificial Pancreas System (DIYAPS). In DIYAPS, commercially available and approved medical devices such as insulin pumps and continuous glucose monitoring sensors are connected and remotely controlled by systems using open-source algorithms to automate insulin delivery. While these systems are cocreated by the DIYAPS community, each user has to build his/her own system and use it at his/her own risk. This includes children and adolescents whose caregivers build and maintain these systems on their behalf.

Initial observational studies have shown significant improvements in glycemic control, quality of life, and sleep quality in adult DIYAPS users [10-12]. A Czech pilot study was the first to report findings in a pediatric population and showed that AndroidAPS (an Android-based DIYAPS) was a safe and feasible alternative to a commercially available system, with predictive low glucose suspension during a winter sports camp [13]. There remains, however, a lack of research examining outcomes of children and adolescents with DIYAPS in everyday life and their social context. This survey assesses the self-reported clinical outcomes of this specific user group.

## Methods

An online survey was distributed to caregivers using DIYAPS through the Facebook groups “Looped” (>11,500 members as of May 2019) and “AndroidAPS users” (>2800 members as of May 2019), other regional subgroups on Facebook, and Twitter. In this context, a caregiver was either a family member or another person who regularly looked after the child or adolescent with diabetes. Demographics and socioeconomic status of the study population were assessed. Participants were also asked for their child’s last three HbA_1c_ measurements and mean time in range (TIR; sensor glucose level between 70 mg/dL or 4.0 mmol/L and 180 mg/dL or 10.0 mmol/L) before and after DIYAPS commencement. In an open-ended question, we asked respondents if they experienced difficulties in making the transition to DIYAPS.

The survey was designed by an interdisciplinary team of medical doctors, social scientists, public health researchers, and patient innovators. Participants were able to choose between two languages (English and German). Data were collected, managed, and analyzed using the secure REDCap electronic data capture tools hosted at Charité - Universitaetsmedizin Berlin [14]. Arithmetic mean, SD, and two-tailed heteroscedastic *t* test were used to perform the statistical analysis. The survey was approved by the Charité ethics committee (EA2/140/18).

## Results

Overall, 209 participants from 21 countries (74.3% from Europe, 12.0% from North America, 6.9% from Asia, and 6.9% from Australia) responded to the survey (Table 1). Of the total, 47.4% children were female, with a median age of 10 years (range: 3-20 years), and 99.4% had type 1 diabetes. The median duration of diabetes was 4.3 (SD 3.9) years, and various types of DIYAPS (AndroidAPS, 48.0%; OpenAPS, 28.4%; Loop, 28.4%; other, 3.4%; and several systems over time, 7.5%) were used. The group had used these systems for a median of 7.5 (SD 10.0) months. Analysis of caregivers’ socioeconomic status indicated that the cohort was evenly distributed across a range of income groups. The responding caregivers’ employment rate was 91.4%, with 58.4% working full-time and 31.8% working part-time. Analysis of the education level showed that 65.2% had an academic or professional degree.

**Table 1:** Demographic data of children and adolescents using Do-it-Yourself Artificial Pancreas Systems, who participated in this survey.

Demographic			n (%)
**Child’s gender**		
	Female		83 (47.4)
	Male		92 (52.6)
**Child’s age (years)**		
	3		6 (3.4)
	4		11 (6.3)
	5		14 (8.0)
	6		14 (8.0)
	7		12 (6.9)
	8		12 (6.9)
	9		15 (8.6)
	10		20 (11.4)
	11		9 (5.1)
	12		19 (10.9)
	13		11 (6.3)
	14		10 (5.7)
	15		12 (6.9)
	16		2 (1.1)
	17		2 (1.1)
	18		2 (1.1)
	20		4 (2.3)
**Child’s type of diabetes**		
	Type 1		174 (99.4)
	Type 2		0 (0.0)
	Other/Unknown		1 (0.6)
**Type of DIYAPS^a^ used**			
	OpenAPS		43 (28.4)
	AndroidAPS		71 (48.0)
	Loop		42 (28.4)
	Other/Unknown		5 (3.4)
**Region (country of residence)**			
	**Europe**		130 (74.3)
		Austria	3 (1.7)
		Bulgaria	9 (5.1)
		Croatia	2 (1.1)
		Czech Republic	12 (6.9)
		Denmark	2 (1.1)
		Finland	8 (4.6)
		France	1 (0.6)
		Germany	46 (26.3)
		Greece	2 (1.1)
		Ireland	3 (1.7)
		Luxembourg	1 (0.6)
		Poland	2 (1.1)
		Slovakia	4 (2.3)
		Spain	3 (1.7)
		Sweden	8 (4.6)
		Switzerland	1 (0.6)
		United Kingdom	23 (13.1)
	**North America**		21 (12.0)
		Canada	5 (2.9)
		United States	16 (9.1)
	**Asia**		12 (6.9)
		South Korea	12 (6.9)
	**Australia/Western Pacific**		12 (6.9)
		Australia	12 (6.9)
**Caregiver’s occupational status**			
	Full-time		101 (58.4)
	Part-time		55 (31.8)
	Unemployed		10 (5.8)
	Retired		0 (0.0)
	Student		2 (1.2)
	Other/Unknown		5 (2.9)
**Caregiver’s household annual net income (US $)**			
	<20,000		19 (12.0)
	20,000-34,999		12 (7.6)
	35,000-49,999		19 (12.0)
	50,000-74,999		33 (20.9)
	75,000-99,999		24 (15.2)
	≥100,000		40 (25.9)

^a^DIYAPS: Do-it-Yourself Artificial Pancreas Systems.

**Figure 1:**
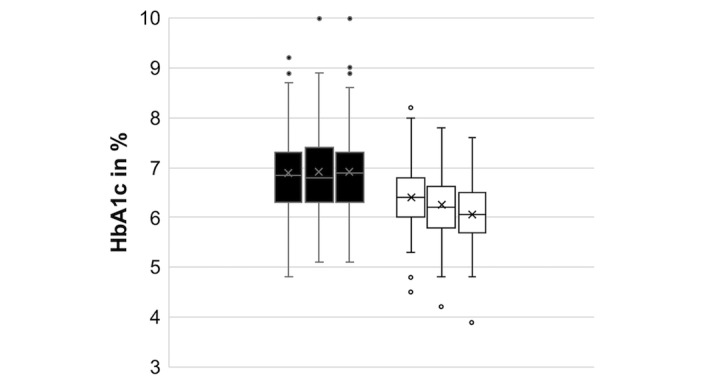
Mean last HbA_1c_ levels of children and adolescents before (black) and after (white) the initiation of Do-it-Yourself Artificial Pancreas Systems. HbA_1c_: hemoglobin A_1c_.

**Figure 2:**
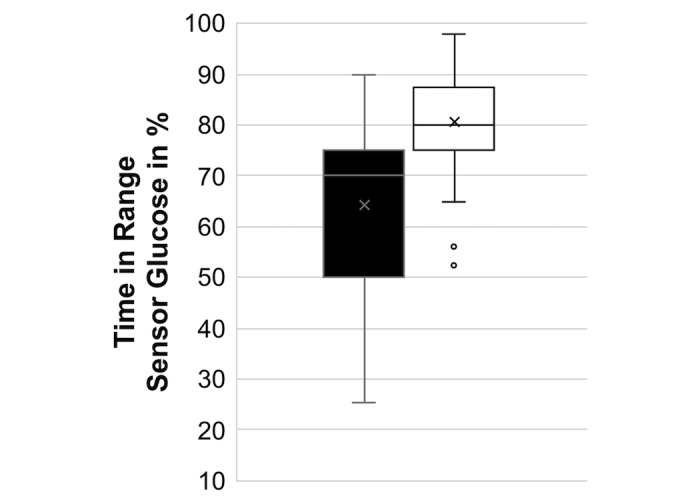
Mean time in range for sensor glucose levels of children and adolescents before (black) and after (white) the initiation of Do-it-Yourself Artificial Pancreas Systems.

On an average, the cohort already had a baseline glycemic control level below the target HbA_1c_ recommended by the International Society for Pediatric and Adolescent Diabetes [11]. Nevertheless, a significant HbA_1c_ improvement of –0.64 percentage points, from a mean HbA_1c_ of 6.91% (SD 0.88%; or 52.0 mmol/mol) to 6.27% (SD 0.67; or 45.0 mmol/mol) after commencing DIYAPS was reported (*P*<.001; Figure 1). The mean TIR increased from 64.2% (SD 15.94%) to 80.68% (SD 9.26; *P*<.001; Figure 2). Participants also reported a continuous HbA_1c_ improvement over time, starting from a mean HbA_1c_ of 6.39% (SD 0.65%; or 46.3 mmol/mol) as their first result after commencement, which equals an improvement of –0.52 percentage points compared to the baseline level, gradually improving to a mean HbA_1c_ of 6.26% (SD 0.69%; or 44.9 mmol/mol) as their second result and a mean HbA_1c_ of 6.06% (SD 0.66%; or 42.7 mmol/mol) as their third result, which equals an improvement of –0.85 percentage points. Users of all DIYAPS systems and all age groups showed similar results (Table 2).

Among the relatively few respondents who indicated difficulties with DIYAPS (n=29), the primary challenge was sourcing the necessary devices and setting up the closed loop. In both cases, successful solutions were primarily found online, although for some, this was a time-consuming process.

**Table 2:** Clinical outcomes in children and adolescents before and after initiation of Do-it-Yourself Artificial Pancreas Systems.

Outcomes and users			Mean (SD)
**All DIYAPS^a^ users**			
	**HbA_1c_^b^**		
		Before	6.91 (0.88)
		After initiation	6.27 (0.67)
	**Time in range**		
		Before	64.2 (15.94)
		After initiation	80.68 (9.26)
**OpenAPS users**			
	**HbA_1c_**		
		Before	7.10 (0.75)
		After initiation	6.36 (0.72)
	**Time in range**		
		Before	67.1 (14.4)
		After initiation	81.7 (7.7)
**AndroidAPS users**			
	**HbA_1c_**		
		Before	6.85 (0.79)
		After initiation	6.24 (0.73)
	**Time in range**		
		Before	63.8 (15.0)
		After initiation	79.5 (7.9)
**Loop users**			
	**HbA_1c_**		
		Before	6.99 (1.00)
		After initiation	6.39 (0.61)
	**Time in range**		
		Before	64.2 (15.4)
		After initiation	79.1 (8.4)
**Children (3-9 years)**			
	**HbA_1c_**		
		Before	6.89 (0.80)
		After initiation	6.31 (0.59)
	**Time in range**		
		Before	66.5 (15.9)
		After initiation	79.2 (8.4)
**Adolescents and young adults (10-20 years old)**			
	**HbA_1c_**		
		Before	6.93 (0.95)
		After initiation	6.23 (0.75)


	**Time in range**		
		Before	62.2 (15.9)
		After initiation	80.1 (9.3)

^a^DIYAPS: Do-it-Yourself Artificial Pancreas Systems.

^b^HbA_1c_: hemoglobin A_1c_.

## Discussion

This survey is currently the largest study of DIYAPS users on a global level and provides new evidence about real-world use of these systems in children and adolescents. Improvement of glycemic control was consistently reported across all pediatric age groups, including adolescents and very young children. Thus, the beneficial effects observed in adult users appear to apply to the pediatric population with no age limitations. These findings are in line with clinical trial results and improvements seen in commercially developed closed-loop systems [5-9].

The occupational and educational level of the responding caregivers was well above the population level; however, the household income levels varied. This finding suggests that DIYAPS may be financially accessible to a variety of socioeconomic groups. Further investigations on the role of all household caregivers’ socioeconomic status and barriers to scaling up use of these systems would be of interest.

Although studies investigating DIYAPS consistently have demonstrated significant improvements in a variety of clinical and patient-reported outcomes, with no accompanying severe adverse events, various stakeholders continue to view the use of DIYAPS with skepticism. Ethical and legal questions have been raised, especially for the vulnerable group of children and adolescents. The off-label use of unregulated medical devices as well as the role of the caregiver taking the decision independently from doctors is the subject of intense debate [15]. Children are dependent on their caregivers’ technological and medical knowledge and skills, both of which are prerequisites of understanding, building, and maintaining a DIYAPS. Moreover, a limited number of diabetes specialists are familiar with DIYAPS and their in-built safety mechanisms. Knowledge is also limited because research focusing on pediatric cohorts have tended to lag behind the adult population. Studies such as this one may therefore help alleviate concerns of health care providers as they are increasingly confronted with caregivers who have opted for DIYAPS for their child’s diabetes management in their day-to-day clinical practice.

This study has several limitations. Outcomes were self-reported by caregivers. Until recently, self-reported data have not been commonly used in clinical research. However, a Norwegian study previously found that self-reported outcomes showed good concordance with data from patient registries reported by health care professionals [16]. Continuous glucose monitoring data were not directly captured in this survey. Therefore, time in hypoglycemia was not assessed. Time spans between HbA_1c_ measurements and TIR as well as DIYAPS versions, settings, and targets might differ individually. With a median DIYAPS experience of 7.5 months, some participants were unable to provide all three HbA_1c_ measurements. With education level and occupational status above the average population level and previous baseline glycemic outcomes below the target, the cohort or DIYAPS community may, in general, not be representative of all families having children with diabetes. To fully evaluate both the benefits and risks of DIYAPS, safety and efficacy trials for all age groups are needed.

The growing #WeAreNotWaiting movement globally is indicative of a paradigm shift whereby traditional, top-down health care solutions are increasingly being complemented by bottom-up and patient-led initiatives. This survey, novel in both its sample size and international scope, provides new evidence that DIYAPS can offer substantial improvements in clinical outcomes for children and adolescents, even in a population that already has achieved glycemic outcomes below the target. However, more research is needed to examine the mechanisms by which these results are achieved; lived experiences of DIYAPS users; adverse events; and what can be learned from this movement in order to accelerate the diffusion of APS technology across the population.

## Abbreviations

DIYAPS: Do-it-Yourself Artificial Pancreas Systems
HbA_1c_: hemoglobin A_1c_
TIR: time in range
